# The Effects of Proresolution of Ellagic Acid in an Experimental Model of Allergic Airway Inflammation

**DOI:** 10.1155/2013/863198

**Published:** 2013-11-26

**Authors:** Claudiney de Freitas Alves, Giovanna Natalia Angeli, Daniely Cornélio Favarin, Edinéia Lemos de Andrade, Javier Emilio Lazo Chica, Lúcia Helena Faccioli, Paulo Roberto da Silva, Alexandre de Paula Rogerio

**Affiliations:** ^1^Laboratório de ImunoFarmacologia Experimental (LIFE), Departamento de Clínica Médica, Instituto de Ciências da Saúde, Universidade Federal do Triângulo Mineiro (UFTM), Rua Manoel Carlos 162, 38025-380 Uberaba, MG, Brazil; ^2^Departamento de Farmacologia, Universidade Federal de Santa Catarina, Florianópolis, SC, Brazil; ^3^Departamento de Análises Clínicas, Toxicológicas e Bromatológicas, Faculdade de Ciências Farmacêuticas de Ribeirão Preto, Universidade de São Paulo (USP), Ribeirão Preto, SP, Brazil

## Abstract

Asthma is a disease of airway inflammation characterized by airway hyperresponsiveness, eosinophilic inflammation, and hypersecretion of mucus. Ellagic acid, a compound derived from medicinal plants and fruits, has shown anti-inflammatory activity in several experimental disease models. We used the classical experimental model, in BALB/c mice, of sensibilization with ovalbumin to determine the effect of ellagic acid (10 mg/kg; oral route) in the resolution of allergic airways response. Dexamethasone (1 mg/kg; subcutaneous route) was used as a positive control. The control group consisted of nonimmunized mice that received challenge with ovalbumin. Ellagic acid and dexamethasone or vehicle (water) were administered before or after intranasal allergen challenge. Ellagic acid accelerated the resolution of airways inflammation by decreasing total leukocytes and eosinophils numbers in the bronchoalveolar lavage fluid (BALF), the mucus production and lung inflammation in part by reducing IL-5 concentration, eosinophil peroxidase (EPO) activity, and P-selectin expression, but not activator protein 1 (AP-1) and nuclear factor kappa B (NF-*κ*B) pathways. In addition, ellagic acid enhanced alveolar macrophage phagocytosis of IgG-OVA-coated beads *ex vivo*, a new proresolving mechanism for the clearance of allergen from the airways. Together, these findings identify ellagic acid as a potential therapeutic agent for accelerating the resolution of allergic airways inflammation.

## 1. Introduction

Asthma is a chronic inflammatory disease that is highly prevalent worldwide and it is characterized by the recruitment of leukocytes, mainly eosinophils, airway hyperreactivity, IgE production, and mucus hypersecretion. The physiopathology of allergic asthma is coordinated mainly by Th2-type immune responses which are characterized by release of cytokines such as interleukin- (IL-) 4, IL-5, and IL-13 and chemokines such as RANTES and CCL11 [1–3]. Most patients with asthma have symptoms that are readily controllable by standard asthma therapies, including *β*2-adrenergic agonists, low doses of inhaled corticosteroids, or leukotriene modifiers [4]. Although these drugs have potent activity, they also have various and severe adverse effects [5, 6]. Therefore, agents of natural origin with very few side effects are required as substitutes for chemical therapeutics. Natural products have long been used in folk medicine as alternative treatment for various diseases, including inflammatory processes of diverse origin. Many medicinal plants provide relief of symptoms comparable to that obtained with allopathic medicines [5, 7, 8]. In the course of an ongoing search for bioactive plant-derived natural products, several groups, including our own, have successfully employed experimental methods to screen plant extracts and plant secondary metabolites for pharmacological activity [9, 10]. Ellagic acid, a polyphenol, is widely found in fruits (e.g., pomegranates, persimmon, raspberries, black raspberries, strawberries, peach, and plumes), nuts (e.g., walnuts and almonds), vegetables, and wine [11, 12]. Ellagic acid is widely known by its antioxidant effects; however, it also demonstrates other biological effects such as anti-inflammatory proprieties [11–13]. Studies from our groups and others have demonstrated the anti-inflammatory activity of ellagic acid and extracts which contain it in the airways [7, 10, 14]. Here, we determined the impact of ellagic acid on the resolution of allergic airways responses.

## 2. Material and Methods 

### 2.1. Materials

Ellagic acid (≤95% of purity—HPLC; Sigma-Aldrich, MO, USA); dexamethasone (Decadron Teuto-Brasileiro, GO, BRA); ovalbumin (Sigma-Aldrich, MO, USA); aluminum hydroxide (Sigma Chemical: Missouri, USA); EDTA (Sigma Chemical: Missouri, USA); isoflurane (anesthetic forane, Abbott: Abbott Park, USA).

### 2.2. Animals

All animal care and procedures used in this study were in compliance with the guidelines on the Use of Animals of the UFTM Ethics Committee (protocol number 162), which follow the NIH “Principles of Laboratory Animal Care” publication no. 85–23. The experiments were conducted using female BALB/c mice (5–7 weeks old and weighing 20–25 g) that were kept in controlled temperature (22 ± 2°C) and humidity (45–55%) under a 12 : 12 h light-dark cycle (lights on 07 : 00 h). Food and water were provided *ad libitum*.

### 2.3. Antigen Immunization, Booster, and Airway Challenge

Mice were sensitized on days 0 and 7, by subcutaneous injection of 10 *μ*g of ovalbumin (Grade III) plus 1 mg of aluminum hydroxide at 0,2 mL of saline. After, sensitization protocol was followed by intranasal challenge (days 14, 15, 16, and 17) with 10 *μ*g of ovalbumin (OVA) in saline and 50 *μ*L of this solution was delivered into the nostrils under isoflurane anesthesia with the aid of a micropipette [15].

### 2.4. Treatment with Ellagic Acid and Controls

Animals were treated with ellagic acid as described by Rogerio et al. [16]. Once ellagic acid demonstrates poor solubility in water the treatment in each animal was carried out with a suspension of ellagic acid in water at dose 10 mg/kg. The suspension was homogenized with the syringe used in the oral administration before each animal treatment [7]. To study the preventive anti-inflammatory effects, mice received ellagic acid (10 mg/kg) or vehicle (water) by gavage 30 minutes prior to intranasal ovalbumin challenge on days 14, 15, 16, and 17 after sensitization. In a second cohort (therapeutic treatment), the mice were treated with ellagic acid (10 mg/kg) or vehicle (water) by oral gavage on resolution phase on days 18, 19, and 20 of the protocol. As a positive control, the mice were treated with dexamethasone as described in the previous schemes (1 mg/kg; subcutaneous route) [16].

### 2.5. Evaluation of the Leukocyte Influx into the Bronchoalveolar Space

On the days 18, 21, or 25 following the inflammation, the mice were euthanised by sodium pentobarbital overdose (70 mg/kg, intraperitoneal), and the BALF and lung were collected. A polyethylene cannula was introduced into the trachea. BALF was performed with 1 mL of phosphate-buffered saline (PBS) plus 0.6 mM methylenediaminetetraacetic acid (EDTA) and placed on ice [16]. The total cell and differential leukocyte counts were made according to Alves et al. [9]. Following centrifugation (400 ×g, 5 min, 4°C), the supernatants of the BALF were collected and stored at −80°C for subsequent cytokine determination. The resolution was quantified by calculating the rate of resolution (time interval required for the number of cell − eosinophils − fall half the maximum value) using the BALF [15, 17].

### 2.6. Histology and Immunohistochemistry

In selected animals, after BALF had been collected, lungs were collected, fixed with 10% phosphate-buffered formalin (v/v), and embedded in paraffin. The histological slides were silanized and the tissues were cut into 5-*μ*m sections, which were then stained with haematoxylin and eosin (H&E) or periodic acid-Schiff (PAS) reagent (Sigma-Aldrich) for light microscopy examination. Scores for peribronchiolar inflammatory cell infiltrates in sections from each lung were graded; a score of 0 indicated the absence of inflammatory cell infiltrates; a score of 1, less than five layers of inflammatory cells in <50% of the bronchiolar submucosa; 2, more than five layers of inflammatory cells in <50% of the bronchiolar submucosa; 3, less than five layers of inflammatory cells in >50% of the bronchiolar submucosa; and 4, more than five layers of inflammatory cells in >50% of the bronchiolar submucosa [18]. The number of PAS staining (PAS+) cells in individual bronchioles was counted as described previously [19].

For histological analyses the lungs were removed, postfixed for 24 h in the same solution, placed in ethanol (70% v/v), and then submerged in paraffin. Tissues embedded in paraffin were cut into thick sections (5 *μ*m). Slides were deparaffinized through a series of xylene baths and rehydrated through graded alcohol solutions. High temperature antigen retrieval was performed by immersion of the slides in a water bath at 95–98°C in 10 mmol/L trisodium citrate buffer pH 6.0, for 40 min. After overnight incubation with primary antibody (goat anti-P-selectin (1 : 1000, Santa Cruz Biotechnology, California, USA)), at 4°C, the slides were washed with PBS and incubated with appropriate biotin-coupled secondary antibody (1 : 250; DakoCytomation, Carpinteria, CA) for 1 h at room temperature. The sections were then washed in PBS and incubated with streptavidin-peroxidase (1 : 250; Invitrogen) for 1 h. The visualization was completed by the use of 3,3′-diaminobenzidine (DAB) (DakoCytomation) in chromogen solution and light counterstaining with Harris's haematoxylin solution (Merck, Darmstadt, Germany). Images were obtained with a microscope (Eclipse 50i; Nikon, Melville, NY) and Digital Sight Camera (DS-Fi1; Nikon). Control and experimental tissues were placed on the same slide and processed under the same conditions. Settings for image acquisition were identical for control and experimental tissues. For each mouse lung, three images were obtained. The images were transferred to a computer, and the average pixel color intensity of P-selectin staining was calculated as described in a previous study [20].

### 2.7. Measurement of Eosinophil Peroxidase (EPO) in the Lung

Eosinophil recruitment in the lung was indirectly measured by means of EPO activity. The lungs were removed and homogenized, and the assays were performed as described by Rogerio et al. [20].

### 2.8. Alveolar Macrophage Isolation and Allergen Clearance

Alveolar macrophages from control or ovalbumin-sensitized and -challenged animals were obtained at therapeutic protocol at day 21 from BALF as described previously by Rogerio et al. [17] and Gilberti et al. [21]. Cells were placed on coverslips in 96-well plates (1.5 × 10^5^ cells/well) in media (RPMI 1640 plus 10% FCS containing L-glutamine and antibiotics) and incubated overnight at 37°C. Nonadherent cells were removed, and the plates were supplemented with fresh medium. Macrophages (from BALF) were treated with ellagic acid (1, 10, or 100 *μ*M), dexamethasone (0.1 *μ*M), or vehicle (DMSO) and incubated in the dark (20 min, 37°C). Ellagic acid was dissolved in dimethylsulfoxide (DMSO, final concentration is 0.1%) and was diluted in 1x PBS to prepare required concentrations. In each treatment, the cells were treated with vehicle or with ellagic acid (1, 10, and 100 *μ*M) or dexamethasone (1 *μ*M) in the presence of alveolar macrophages [22]. Rabbit anti-ovalbumin IgG-Ab-coated polybead microsphere beads were prepared (according to the manufacturer's instructions (Polysciences)) and added to the cells at a ratio of 13 beads/cell. Immediately (time 0) or after 15 min, cells were washed with PBS and paraformaldehyde (4%) was added. After 30 min, cells were washed again with PBS, and FITC-conjugated goat anti-rabbit Ab (1 : 150) was added (35 min, room temperature, in the dark). Supernatants were removed, and, after washing in PBS, the cells on the coverslips were mounted for fluorescent microscopy. Beads were counted in both light and fluorescence images that were acquired for 50 cells in each incubation. Because Abs are not membrane permeable, only adherent noninternalized beads are fluorescent. This allows for distinction between internalized and cell-adherent beads. To quantify particle internalization, the number of surface-bound beads was counted from the fluorescence images and the total number of beads from the nonfluorescent images. The phagocytosis index was determined by subtracting the number of fluorescent beads from the total number of beads (nonfluorescent images) to derive the number of internalized beads. For each cell counted, the number of internalized beads was divided by the total number of beads to derive its phagocytosis index [17].

### 2.9. Statistical Analysis

The data were reported as the means ± SEM. The means from the different treatments in each individual experiment were compared by ANOVA. When significant differences were identified, the individual comparisons were subsequently made with the Tukey's test. Values of *P* < 0.05 were considered statistically significant.

## 3. Results

### 3.1. The Preventive Effect of Ellagic Acid on Leukocyte Recruitment to the BALF

**Figure 1:**
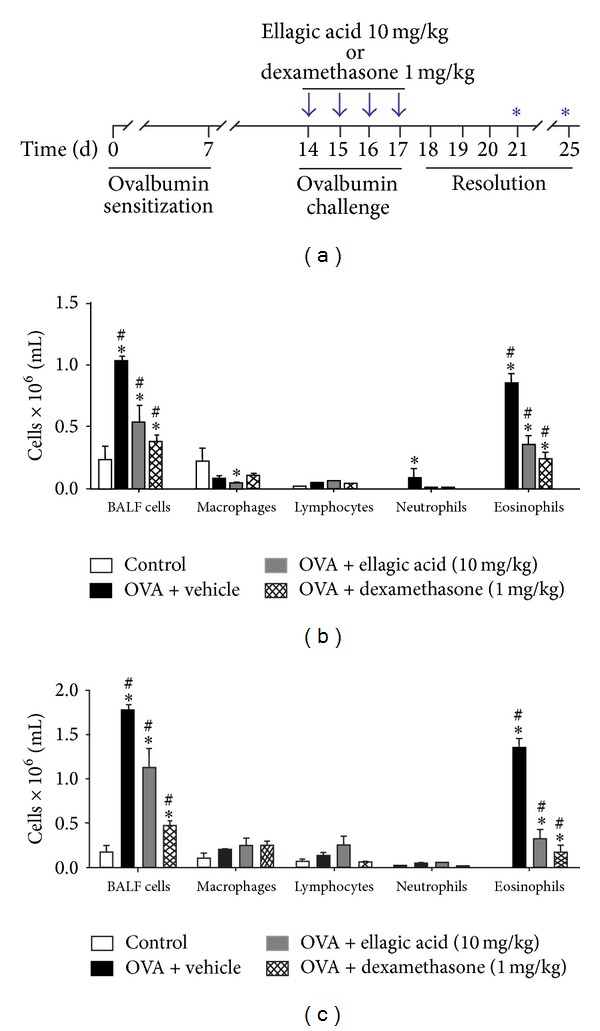
Ellagic acid prevents the development of airway inflammatory responses in experimental airways allergic inflammation. Mice received ellagic acid (10 mg/kg, p.o.), dexamethasone (1 mg/kg, s.c.), or vehicle (water, p.o.) 30 minutes prior in accordance to the preventive protocol (see Section 2) and euthanized on 21 or 25 days and cells in bronchoalveolar lavage were counted (a). BALF cells and leukocytes subset at 21 days (b); BALF cells and leukocytes subset at 25 days (c). One group of animals received vehicle (control group). Results represent the mean ± SEM, of two or more independent experiments with four mice per group per experiment. *P < 0.05 compared with control group; ^#^ *P* < 0.05 compared with ovalbumin + vehicle group.

We first evaluated the preventive effect of ellagic acid (10 mg/kg) on the resolution of allergic airways inflammation. So, ellagic acid was given to sensitized animals 30 min prior to each daily allergen intranasal challenge for a period of 4 d (see Section 2) (Figure 1(a)). In all time points analyzed (days 18 (peak of inflammation; data not shown), 21 (Figure 1(b)), and 25 (Figure 1(c))), the numbers of BALF total cells, eosinophils, and/or macrophages, but not lymphocytes, from the vehicle-treated group were significantly increased compared to the control group. In agreement with previous studies [16] we confirmed the anti-inflammatory effect of ellagic acid in the peak of inflammation (day 18) (data not shown). We extend this result and we demonstrate that ellagic acid accelerated the resolution of allergic airways inflammation (at day 21). Ellagic acid or dexamethasone significantly reduced the total leukocytes number ~50% from 1.03 ± 0.04 (vehicle) (mean × 10^6^/mL ± SEM) to 0.53 ± 0.14 (ellagic acid) or 0.37 ± 0.06 (dexamethasone) (Figure 1(b)). The BALF eosinophil numbers of mice treated with ellagic acid were also reduced by approximately ~60% from 0.86 ± 0.07 (vehicle) to 0.34 ± 0.08 (ellagic acid), while the dexamethasone treatment reduced by ~70% (0.23 ± 0.05) (Figure 1(b)). In addition, BALF neutrophils were also reduced from 0.09 ± 0.07 (vehicle) to 0.01 ± 0.00 (ellagic acid) while no neutrophils were counted in dexamethasone treatment. Moreover, the BALF macrophages were significantly reduced in ellagic acid treatment from 0.22 ± 0.11 (vehicle) to 0.05 ± 0.01 (ellagic acid) (Figure 1(b)). No difference of lymphocytes numbers among the groups was observed. The results from day 25 (Figure 1(c)) were similar to day 21. Ellagic acid and dexamethasone reduced the number of total leukocytes of 0.08 ± 1.76 (vehicle) to 1.12 ± 0.23 (ellagic acid) (mean × 10^6^/mL ± SEM) and 0.48 ± 0.05 (dexamethasone). In addition, the eosinophils numbers in BALF were reduced also from 1.37 ± 0.08 (vehicle) to 0.33 ± 0.12 (ellagic acid) and 0.16 ± 0.09 (dexamethasone) (Figure 1(c)). No difference of lymphocytes, neutrophils, and macrophages numbers among the groups was observed.

### 3.2. The Effect of Ellagic Acid on Resolution of Airways Inflammation

**Figure 2:**
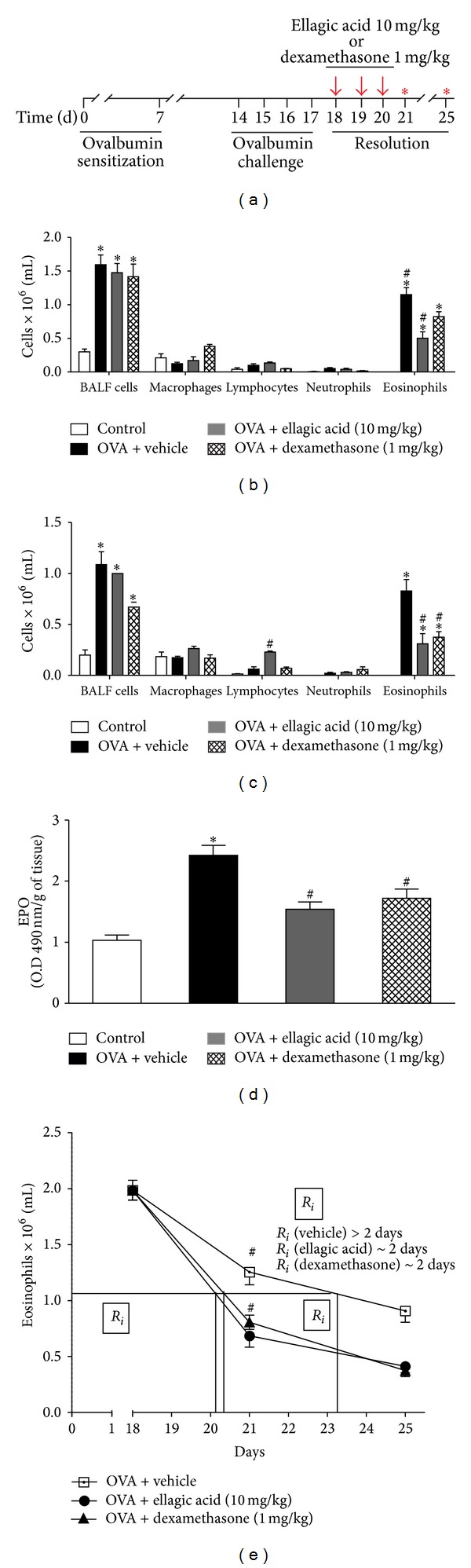
Ellagic acid demonstrates therapeutic anti-inflammatory activity on the resolution phase in the experimental airways allergic inflammation model. Mice received ellagic acid (10 mg/kg, p.o.), dexamethasone (1 mg/kg, s.c.), or vehicle (water, p.o.) after ovalbumin challenge at 18, 19, and 20 days (see Section 2) and were euthanized on 21 or 25 days and cells in bronchoalveolar lavage were counted (a). BALF cells and leukocytes subset at 21 days (b); BALF cells and leukocytes subset at 25 days (c). One group of animals received vehicle (p.o.) only (control group). EPO concentration in the lung at 21 day (d). The resolution interval (*R*_*i*_) (e). Results represent the mean ± SEM, of two or more independent experiments with four mice per group per experiment. *P < 0.05 compared with control group; ^#^ *P* < 0.05 compared with ovalbumin + vehicle group.

In the view of ellagic acid's protective actions in the airways, we next evaluated the influence of ellagic acid on the resolution of established airway inflammation. After animals were ovalbumin-sensitized and intranasal-challenged, ellagic acid, dexamethasone, or vehicle was administered for 3 consecutive days (protocol days 18–20), and no further allergen challenges were performed (Figure 2(a)). BALF leukocytes were enumerated on days 21 and 25. In both days, the numbers of BALF total cells, eosinophils, macrophages, and lymphocytes from the vehicle-treated group were significantly increased compared to the control group (Figures 2(b) and 2(c)). Ellagic acid or dexamethasone decreased the BALF eosinophil numbers at day 21 about 70% from 1.31 ± 0.11 (vehicle) to 0.39 ± 0.07 (ellagic acid) or 0.82 ± 0.07 (~37%) (dexamethasone) (mean × 10^6^/mL ± SEM). No significant difference was observed in the neutrophils, lymphocytes, and macrophages number of animals treated with ellagic acid or dexamethasone compared to vehicle-treated group (Figure 2(b)). At day 25, ellagic acid and dexamethasone treatment decreased eosinophil numbers in the BALF (Figure 2(c)). In this point, BALF eosinophils were decreased by *∼*80% from 1.10 ± 0.12 (vehicle) to 0.23 ± 0.05 (ellagic acid) or 0.38 ± 0.05 (~65%) (dexamethasone). Ellagic acid, different from dexamethasone, increased the number of lymphocytes from 0.10 ± 0.02 (vehicle) to 0.32 ± 0.03 (ellagic acid) (Figure 2(c)). No significant difference was observed in the neutrophils and macrophages number of animals treated with ellagic acid or dexamethasone compared to vehicle-treated group.

We next determined the influence of ellagic acid on the resolution of established eosinophilic airway inflammation. Resolution interval (*R*_*i*_) is defined as the time required for the cell numbers to decrease to 50% of the maximum at peak inflammation (the interval between peak of inflammation, at 18th day) [17]. In vehicle-exposed mice, the endogenous resolution interval for BALF eosinophils was *∼*5 days (Figure 2(d)). Ellagic acid, similar to dexamethasone, displayed an important decrease on resolution interval for BALF eosinophils (*∼*2 days to *∼*50% of the resolution interval) compared to vehicle (Figure 2(d)), indicative of more rapid resolution of allergic airway inflammation.

We also determined the effect of ellagic acid on eosinophil peroxidase (EPO) activity in the lung at day 21. In the time point analyzed, the EPO activity from the vehicle-treated group was significantly increased compared to the control group (Figure 2(e)). Ellagic acid and dexamethasone reduced EPO activity from 2.42 ± 0.16 (vehicle) to 1.54 ± 0.11 (ellagic acid) or 1.72 ± 0.15 (dexamethasone) (Figure 2(e)).

### 3.3. Ellagic Acid Accelerates the Resolution of Lung Inflammation and Airway Mucus Metaplasia

**Figure 3:**
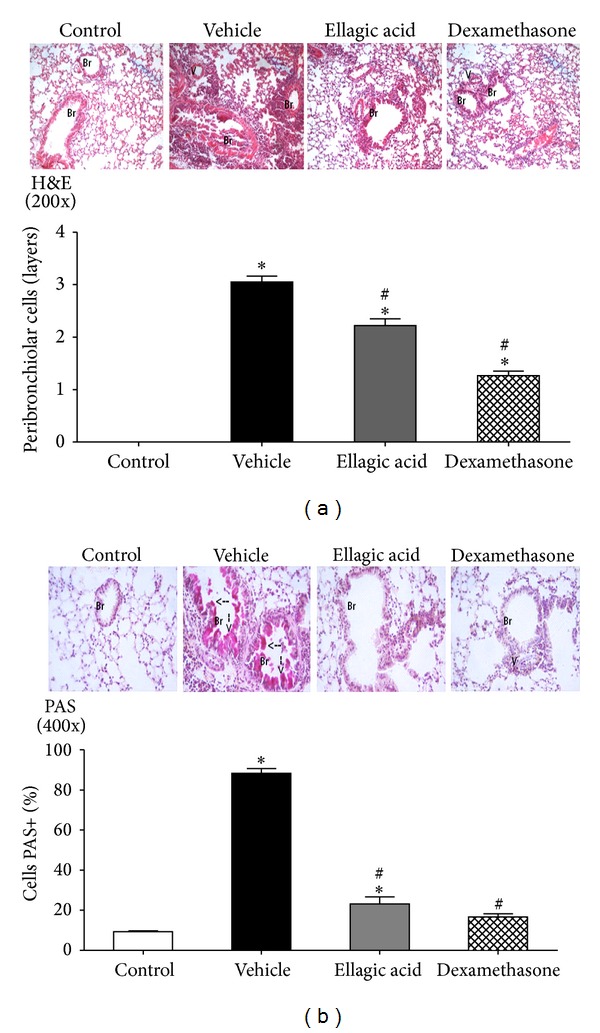
Effect of the therapeutic treatment with ellagic acid on lung inflammation and mucus secretion. Mice were treated with ellagic acid (10 mg/kg) or dexamethasone (1 mg/kg) or vehicle (water). One group of animals received vehicle (control group). Lung tissue sections were obtained at protocol day 21 and stained with H&E (original magnification: 200x) and the inflammation was quantified (a). Lugs tissue sections were also stained with PAS reagent (original magnification: 400x) and bronchial PAS-positive cells were quantified (b). Arrows indicate examples of mucus-containing (magenta) goblet cells. Results represent the mean ± SEM of two or more independent experiments with three mice per group. *P < 0.05 compared with control group; ^#^ *P* < 0.05 compared with ovalbumin + vehicle group. Br: bronchus and V: venule.

The proresolving actions of ellagic acid were also evident for lung inflammation and airways mucus metaplasia. The lungs of the vehicle-treated mice demonstrated increased edema, thickening of the alveolar septum and interstitium, and leukocyte infiltration compared to the control group (Figures 3(a) and 3(b)). In addition, vehicle-treated mice demonstrated increase of mucus production (Figure 3(a)). Ellagic acid and dexamethasone-treatment reduced all of the aforementioned inflammatory parameters compared to the vehicle-treated mice, especially leukocyte infiltration. Ellagic acid and dexamethasone treatment reduced the airways inflammation by ~30% or ~60%, respectively (Figures 3(a) and 3(b)). In addition, ellagic acid and dexamethasone also decreased the amount of mucus secretion by 80% and 90%, respectively, as indicated in arrows (Figure 3(a)).

### 3.4. Effect of Ellagic Acid on P-Selectin, AP-1, and NF-*κ*B Expression on the Lung and IL-5 Concentration in BALF

**Figure 4:**
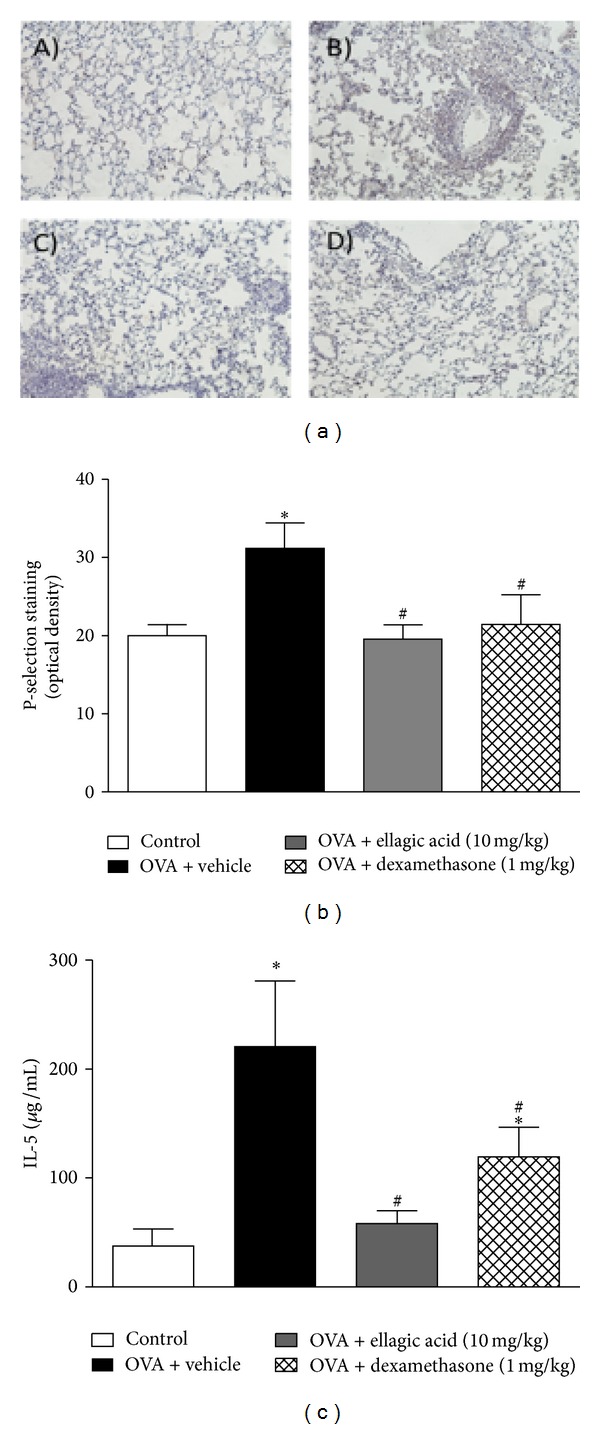
Effect of ellagic acid on P-selectin expression in the lung and IL-5 concentration in the BALF. Representative images of P-selectin immunohistochemistry staining of (A) control; (B) OVA + vehicle; (C) OVA + ellagic acid; (D) OVA + dexamethasone (a). The mean intensity of P-selectin staining was determined from image analysis and represented as arbitrary units (b). The analyses of lung ((a) and (b)) and IL-5 concentration in the BALF (c) were carried out in the resolution phase (at 21 days). Values represent the mean ± SEM (*n* = 4 per group). *P < 0.05 compared with control group; ^#^ *P* < 0.05 compared with ovalbumin + vehicle group.

In order to assess the effects of ellagic acid on the resolution phase (at day 21) we evaluated the P-selectin, NF-*κ*B, and AP-1 expression in the lung. Constitutive P-selectin expression was observed in control mice. In the vehicle-treated and ovalbumin-immunized and -challenged group, we observed an upregulation of P-selectin expression along the bronchial epithelium compared to control group. Of note, ellagic acid and dexamethasone treatment significantly reduced the expression of P-selectin in the lung (by 35% and 33%, resp.) compared to the vehicle-treated animals (Figures 4(a) and 4(b)). No significant alterations were observed in p65 NF-*κ*B or AP-1 among the groups (data not shown).

We next evaluated the therapeutic effect of ellagic acid on the BALF cytokine levels at 21 days. The concentrations of IL-5 were increased in the vehicle-treated mice compared to control mice (Figure 4(c)). Ellagic acid and dexamethasone reduced the IL-5 concentration *∼*88% from 220 ± 60 *µ*g/mL (vehicle) to 58 ± 11 *µ*g/mL (ellagic acid) and 119 ± 27 *µ*g/mL (~55%) (dexamethasone) (mean ± SEM), respectively (Figure 4(c)). No significant differences or detections were observed on IL-10, IL-17, and IFN-*γ* concentration among the groups or when compared to the vehicle-treated group control (data not shown).

### 3.5. Ellagic Acid Enhances Macrophage Clearance of Allergen by Phagocytosis

**Figure 5:**
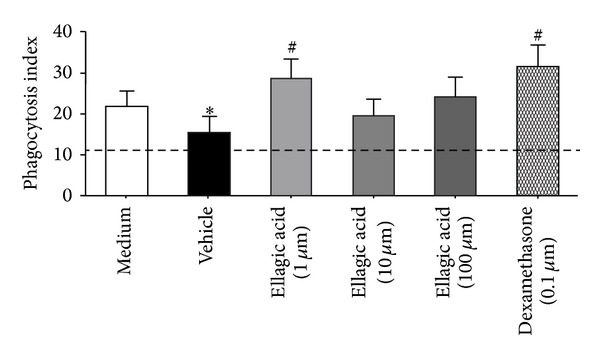
Ellagic acid increased macrophage phagocytosis of allergen. BALF cells on protocol day 21 (see Section 2), from OVA sensitized and challenged (*ex vivo*), were used to determine the phagocytosis index using rabbit anti-OVA IgG-coated beads (2 mm) that are detectable by light microscopy. In nonpermeabilized cells, a fluorophore-tagged Ab (FITC-conjugated goat anti-rabbit Ab) was used to distinguish adherent (fluorescent) from internalized (nonfluorescent) beads (original magnification: 1000x). A phagocytosis index was calculated after 15 min in the presence of ellagic acid (1, 10, and/or 100 *μ*M), dexamethasone (0.1 *μ*M), vehicle, or medium (controls). Results represent the mean ± SEM of two or more independent experiments with three mice per group per experiment. *P < 0.05 compared with control (medium); ^#^ *P* < 0.05 compared with vehicle group.

We used macrophages from BALF mice that were ovalbumin-sensitized and intranasal-challenged (*ex vivo*) of protocol at day 21 to evaluate the phagocytosis. Ellagic acid (1 *μ*M), similar to dexamethasone, increased the macrophage phagocytosis within 15 min (37°C) for allergen-coated beads (Figure 5).

## 4. Discussion

In the few decades, the number of the new drugs discovered that originated from nature has increased considerably [9, 10]. The current therapy for the treatment of airway inflammation has not changed to the same degree. Inhaled corticosteroids and *β*2-adrenoceptor agonists remain as the mainstay of asthma treatment. Although these medicines are effective they demonstrated significant side effect for the long use [6]. Thus, the identification of new molecules that are able to prevent or treat inflammatory airway diseases is highly desirable. Like other polyphenols, ellagic acid demonstrates a wide range of biological activities, which suggest that they could have beneficial effects on human health. Ellagic acid has antioxidant, anti-inflammatory, and other activities [13]. Several studies have demonstrated that ellagic acid possesses anti-inflammatory properties in the airways [7, 10, 23]. In the present work we extend previous results and highlight for the first time the effect of ellagic acid on the resolution of airway inflammation in an airways allergic inflammation experimental model. Ellagic acid dampens the inflammation, mainly the eosinophilic inflammation, as in the BALF and in the lung and reduced the mucus production. These effects could be associated to reduction of IL-5 concentration in the BALF and P-selectin expression in the lung.

Phytochemical and pharmacological studies have identified many potential anti-inflammatory substances, especially those derived from plants used in folk medicine. *Lafoensia pacari* (Lythraceae) has been used in traditional medicine to treat gastric ulcers and inflammation in the state of Mato Grosso (Brazil). In a bioassay-guided fractionation of the *Lafoensia pacari* extract, Rogerio et al. [10] identified the ellagic acid as the compound responsible for the reduction of eosinophils recruitment into the peritoneal cavity of mice induced to injury by *Histoplasma capsulatum*-derived *β*-glucan. The reduction in the recruitment of eosinophils to the BALF by ellagic acid has also been observed in an ovalbumin-induced experimental allergic airway inflammation [16]. In addition, *Lafoensia pacari* extract and ellagic acid demonstrated also antiedematous activity in a mouse paw edema model in mice [10].

Ellagitannins present in the pomegranate (*Punica granatum* L.) fruit, which has been used for centuries for medical purposes, are hydrolysed to ellagic acid in the gut and are then metabolised by the colonic microflora to form urolithins (A and B) [24, 25]. Interestingly, these ellagic acid metabolites, urolithins, have also demonstrated anti-inflammatory effects, which could potentially promote synergic effect with ellagic acid [26, 27]. Studies with pomegranate extract have demonstrated its anti-inflammatory effects in murine models of collagen-induced arthritis [28] and murine model experimental colitis [29, 30]. In an experimental model of acute lung injury (ALI) (LPS initiated), the pomegranate extract also reduced the myeloperoxidase (a heme enzyme present in the primary granules of polymorphonuclear leukocytes neutrophils) in the lungs of mice [31], suggesting that ellagic acid might be involved in this process. In fact, our group demonstrated the anti-inflammatory effects of ellagic acid in HCl acid-initiated ALI [7].

In asthma, a complex network of cytokines and chemokines mediates the inflammatory response. IL-5 is essential for eosinophil migration from the bone marrow to the blood [32] and specifically supports terminal differentiation and proliferation of eosinophil precursors as well as activating mature eosinophils [33]. In the resolution phase, ellagic acid accelerated the resolution of eosinophilic inflammation by reducing the eosinophils number in the BALF and in the lung as well as eosinoperoxidase (EPO) activity in the lung. These effects could be associated with reduction of IL-5 concentration in the BALF.

There is a great amount of evidence indicating that adhesion molecules are critically involved in leukocyte control; we next evaluated the expression of P-selectin in the lungs, which is also considered to be an important target for modulating eosinophilic influx to the inflammatory tissue [34, 35]. Our findings revealed that the ellagic acid consistently decreased the expression of P-selectin in the bronchial epithelium of ovalbumin-immunized and -challenged mice. Therefore, the reported inhibition of P-selectin expression is expected to contribute to anti-inflammatory actions in synergism of inhibition of IL-5.

In the setting of allergen-driven inflammation, inhaled allergen needs to be cleared to facilitate resolution of inflammation [17, 36]. Ellagic acid increased alveolar macrophage phagocytosis of allergen-coated beads *in vitro* without a concentration-dependent manner. Ellagic acid promoted increased allergen engulfment by alveolar macrophages *in vitro*. Therefore, ellagic acid demonstrated proresolving mechanism for allergic airway responses.

In conclusion, these results demonstrate protective anti-inflammatory and proresolving actions for ellagic acid in airway inflammation. Ellagic acid decreased eosinophil recruitment and airway mucus metaplasia; moreover it promoted the resolution of allergic airway enhancing allergen clearance. Together, these results point ellagic acid as a therapeutic candidate to treat airways diseases such asthma.
